# Models Predicting Postpartum Glucose Intolerance Among Women with a History of Gestational Diabetes Mellitus: a Systematic Review

**DOI:** 10.1007/s11892-023-01516-0

**Published:** 2023-06-09

**Authors:** Yitayeh Belsti, Lisa Moran, Demelash Woldeyohannes Handiso, Vincent Versace, Rebecca Goldstein, Aya Mousa, Helena Teede, Joanne Enticott

**Affiliations:** 1 Monash Centre for Health Research and Implementation, Faculty of Medicine, Nursing and Health Sciences, Monash University, Melbourne, Australia; 2 Deakin Rural Health, School of Medicine, Deakin University, Warrnambool, Australia; 3 Monash Health, Clayton, Melbourne Australia

**Keywords:** Glucose intolerance, Prognostic model, Predictive model, Prognosis, Gestational diabetes mellitus, T2DM

## Abstract

**Purpose of Review:**

Despite the crucial role that prediction models play in guiding early risk stratification and timely intervention to prevent type 2 diabetes after gestational diabetes mellitus (GDM), their use is not widespread in clinical practice. The purpose of this review is to examine the methodological characteristics and quality of existing prognostic models predicting postpartum glucose intolerance following GDM.

Recent Findings.

A systematic review was conducted on relevant risk prediction models, resulting in 15 eligible publications from research groups in various countries. Our review found that traditional statistical models were more common than machine learning models, and only two were assessed to have a low risk of bias. Seven were internally validated, but none were externally validated. Model discrimination and calibration were done in 13 and four studies, respectively. Various predictors were identified, including body mass index, fasting glucose concentration during pregnancy, maternal age, family history of diabetes, biochemical variables, oral glucose tolerance test, use of insulin in pregnancy, postnatal fasting glucose level, genetic risk factors, hemoglobin A1c, and weight.

**Summary:**

The existing prognostic models for glucose intolerance following GDM have various methodological shortcomings, with only a few models being assessed to have low risk of bias and validated internally. Future research should prioritize the development of robust, high-quality risk prediction models that follow appropriate guidelines, in order to advance this area and improve early risk stratification and intervention for glucose intolerance and type 2 diabetes among women who have had GDM.

**Supplementary Information:**

The online version contains supplementary material available at 10.1007/s11892-023-01516-0.

## Introduction

Gestational diabetes mellitus (GDM) is a condition where women without a previous diagnosis of diabetes exhibit abnormal blood glucose levels during pregnancy [1, 2]. It is one of the most common pregnancy complications worldwide [3, 4], affecting up to 14 million women annually [5, 6]. The prevalence of GDM is increasing globally, due to changes in lifestyle, increasing rates of maternal obesity [7–9], and evolving diagnostic criteria. In 2021, according to the International Diabetes Association, the estimated pooled standardized prevalence of GDM globally was 14.0%, and regionally was 27.6% in the Middle East and North Africa, 20.8% in South-East Asia, 14.7% in Western Pacific, 14.2% in Africa, 10.4% in South America and Central America, 7.8% in Europe, and 7.1% in North America and the Caribbean [2].

Often, blood glucose levels associated with GDM will become normal after delivery; however, these women remain at high risk of developing postpartum metabolic abnormalities such as glucose intolerance and type 2 diabetes mellitus (T2DM). According to recent literature, 12.3 to 60.0% of pregnant women who had GDM will develop some form of glucose intolerance up to 15 years postpartum [10–14] which increases to 70.0% 28 years after pregnancy [11, 15], although this varies in different populations and ethnic groups. Hence, women with a history of GDM have a greater than sevenfold risk of developing postpartum glucose intolerance than those who were normoglycemic [5, 16]. For this reason, the American College of Obstetricians and Gynecologists (ACOG) and the American Diabetes Association (ADA) recommend postpartum screening of all mothers who had GDM from 4 to 12 weeks postpartum for timely intervention [17, 18].

Previous studies have reported a range of prognostic factors associated with risk of developing postpartum glucose intolerance after GDM, which include demographic and clinical factors, antepartum laboratory results, and metabolic factors. For example, factors including age, increased parity, higher pre-conception body mass index (BMI), family history of diabetes, insulin therapy during pregnancy, degree of hyperglycemia during pregnancy (higher area under the curve (AUC) of glucose, higher fasting plasma glucose (FPG)), and impaired pancreatic β-cell function were consistently found to be associated with postpartum glucose intolerance [11, 13, 19–23]. Abnormal findings on a variety of antepartum glucose tolerance tests (OGTT) [24–28] were also reported to be associated with a high risk of post-partum glucose intolerance (e.g., low insulinogenic index II levels on the antepartum 75-g OGTT (42)). More than 60 genetic factors have been identified in association with T2DM. Given women with GDM have a family history of T2DM, it has been found that some genetic variants of T2DM are also associated with early or late postpartum glucose intolerance among women who had GDM [29–33]. In addition, a range of specific metabolic biomarkers including amino acids (branched-chain amino acids, hexose), lipids (linoleic acid, phospholipids, lysophosphatidylcholines, acylcarnitines, sphingomyelins (i.e., SM (OH) C14:1)), *p-cresol* sulfate, and glycocholic acid have also been reported as predictive for postpartum glucose intolerance among women who had GDM [34–38].

Risk prediction models have the advantage of identifying women who are at high risk of developing glucose intolerance after GDM with greater accuracy than single markers and in a timely manner (e.g., years before development). This enables women and their healthcare providers to ensure ongoing screening and implementation of early prevention strategies to optimize health outcomes. Risk prediction models can be applied with each women at any time, which can be important as it has been shown that the majority of women (even those at high risk of postpartum T2DM) did not attend postpartum screening for glucose intolerance [39, 40]. Since glucose intolerance is well known to be effectively managed with lifestyle modification [41, 42], early identification of these at-risk women and more focused ongoing screening may prevent T2DM.

Prediction models that have strong predictive ability, validated, generalizable, and based on easily accessible variables, is required in order to effectively prevent postpartum T2DM risk. A systematic review is needed to aid clinicians in selecting postpartum T2DM risk prediction tools and to summarize all available prediction models for researchers. Therefore, this systematic review aims to summarize and critically evaluate the reporting quality, methodological characteristics, and risk of bias of studies reporting prediction models for developing postpartum glucose intolerance developing after GDM.

## Methods

This study was conducted according to the Preferred Reporting Items for Systematic Reviews and Meta-Analyses (PRISMA) [43] and using The Checklist for critical Appraisal and data extraction for systematic Reviews of prediction Modelling Studies (CHARMS) checklist [44]. The protocol for this systematic review is registered at the international prospective register of systematic reviews (PROSPERO); CRD42022327239.

### Formulating the Review Question and Protocol

The review question was formulated based on the PICOTS framework (population, intervention, comparison, outcome, time, and settings) as recommended by the CHARMS checklist. The study protocol was developed by considering the rationale, objectives, design, methodology, and statistical considerations of the systematic review (Table 1).

**Table 1:** PICOTS of the review question

Domain	Description
Population	The population of interest comprises women who had gestational diabetic mellites
Intervention (model)	The focus is on the prognostic models predicting postpartum glucose intolerance among women who had gestational diabetes mellitus
Comparator	The existence of alternative models was not considered in our case study
Outcome(s)	The outcome was defined as postpartum glucose intolerance
Timing	Here, we focus on development of postpartum glucose intolerance until 20 years of delivery
Setting	Available prediction models for the prediction of postpartum glucose intolerance among women who had gestational diabetes mellitus in all settings (such as hospitals, primary care, secondary, tertiary settings, and community-based) were included

### Main outcome(s)

The main outcome of interest for this study is the development of glucose intolerance in women with a history of GDM. This encompasses metabolic conditions such as T2DM, pre-diabetes (impaired fasting glucose (IFG) and impaired glucose tolerance (IGT)) which developed within 20 years postpartum. These are defined and identified by fasting plasma glucose concentrations and/or OGTT results according to the World Health Organization criteria [45], national or regional Diabetes Association diagnostic criteria, or specified local criteria. Patients with IFG and/or IGT are now referred to as having “pre-diabetes” indicating the relatively high risk for the development of T2DM in these patients.

### Eligibility Criteria

Prediction models were conducted to predict the risk of postpartum glucose intolerance among women who had GDM worldwide in all settings including hospitals, primary care, secondary, tertiary, and community-based settings. We included both prospective and retrospective cohort prognostic model studies. We did not restrict studies by ethnic origin or parity.

Studies with no original data (meetings, editorials, letters, narrative reviews, and commentaries), studies that were performed with a cohort of women with T2DM before pregnancy, and studies that were published in languages other than English were excluded.

### Search Strategy and Screening

The search was conducted on May 21, 2022 across eight databases: Ovid Medline, Ovid Embase, Ovid Emcare, Scopus, Web of Science, CINHAL, Maternity & Infant Care Database (MIDIRS), and Global Health 1910 to 2022 Week 18 attached in Appendix S1. We also manually searched the references of the selected articles to identify additional eligible studies.

Studies identified on database searching were imported to Covidence web-based software (developed by Australian not-for-profit company called SaaS enterprise) for the title and abstract screening. Title and abstract screening and full-text reviews were done by two independent reviewers (YB and DH) based on the aforementioned eligibility criteria, and disagreement was resolved by discussion.

### Critical Appraisal

The CHARMS tool was applied to assess the methodological quality and relevance of studies. The source of data used for prediction model development was assessed. Participants’ selection (method, setting, inclusion, and exclusion criteria); definition, clarity, consistency of outcome of interest, and candidate predictors’ assessment used; sample size used for prediction model development; missing data handling; methodologies used for model development, performance measurement, and model evaluation; and interpretation of the results were assessed by the checklist.

### Risk of Bias (Quality) Assessment

Two researchers (YBM, DWH) assessed the risk of bias. In abstract and full-text screening, discrepancies were resolved by discussion, and consensus was reached on all discrepancies. Assessment of risk of bias and model applicability was conducted using the Prediction models Risk Of Bias Assessment Tool (PROBAST) tool. This involves the assessment of four domains (participants, predictors, outcome, and analysis) to cover key aspects of prediction model studies. Under these four domains, there are 20 signaling questions overall. These questions were scored as “Low,” “High,” or “Unclear.” A low score indicates a low risk of bias, whereas high shows the presence of bias, and unclear was used when there was insufficient reported information to decide on risk. The overall risk of bias was graded as low risk when all domains were considered low risk, and high risk when at least one of the domains was considered high risk. The first three domains (participants, predictors, outcome) were also rated for concern regarding applicability (low, high, or unclear) to the systematic review question. Concerns regarding applicability were rated similarly to risk of bias, but without signaling questions (Table S1).

### Data Extraction (Selection and Coding)

A data extraction grid was created including all relevant variables and key elements in Transparent Reporting of a multivariable prediction model of Individual Prognosis Or Diagnosis (TRIPOD) checklist which was pilot tested by using sample articles and modified accordingly. The following variables were extracted by two authors independently: country, source of data, participants (ethnicity, maternal characteristics), outcomes to be predicted, candidate predictors (index tests), sample size, missing data, model development, model performance measurements (calibration discrimination), model evaluation, and results (Appendix S2).

### Strategy for Data Synthesis

Data synthesis was performed using thematic and context analysis to summarize the methodologies used to develop the prediction studies, participant selection, predictor variable selection and collection, outcome determination, analysis used for model development, variables included in the final model, and performance measures used. Appropriate data was presented in the form of summary tables and, where relevant, graphical representations of the data. Where there was a lack of homogeneity in methods used to develop the prediction models and different sets of predictors used to develop different prediction models, meta-analysis was not performed as merging these models may lead to highly correlated data and inflated estimates [46, 47]. If meta-analysis was not indicated, then qualitative evaluation and synthesis of estimates were applied to summarize and appraise the available model estimates.

## Results

### Main Characteristics of Included Studies

**Fig. 1:**
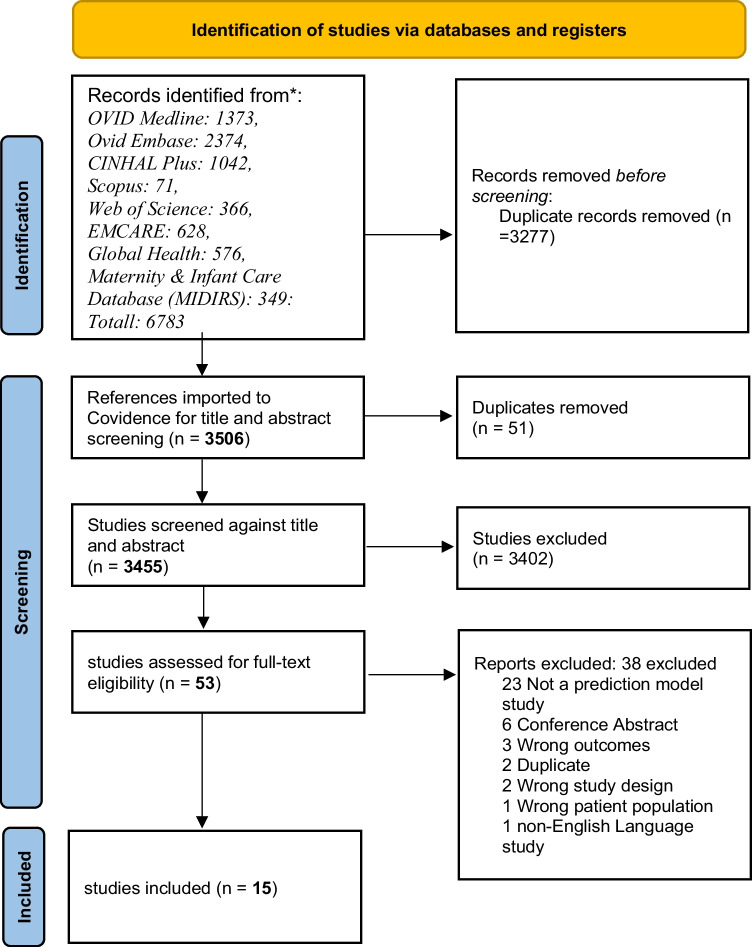
PRISMA flow diagram showing the systematic review process

The systematic review process is presented on the flow chart in Fig. 1. The electronic search method yielded 3455 unique articles, of which 3402 articles were excluded on title and abstract screening leaving 53 studies to be assessed by full text. Following full-text review, a further 38 articles were excluded. Finally, fifteen studies reporting 15 risk prediction models were identified and included in this review. All included studies were model development studies, with no external validation studies found. Included models were developed in six different countries or regions: four in the USA [35, 48••, 49••, 50], six in Europe [24, 36, 51, 52••, 53, 54], two in Australia [37, 55••], and one in Asia [56], Canada [57], and Ethiopia [58••] between 1995 and 2022.

The primary outcomes of included studies were reported as follows: T2DM (*n* = 10) [35–37, 49••, 50, 51, 53, 55••, 56, 57], glucose intolerance (*n* = 4) [24, 52••, 54, 58••], and IGT (*n* = 1) [48••]. Muche et al. [58••] diagnosed glucose intolerance as postpartum pre-diabetes (IFG: FPG 100–125 mg/dL; IGT: 2-h plasma glucose in 75 g OGTT 140–199 mg/dL) or diabetes (FPG > 126 mg/dL, or 2-h plasma glucose > 200 mg/dL in OGTT or random plasma glucose > 200 mg/dL) (Table 2). Postpartum diagnosis of T2DM/prediabetes for Bartakova et al. [59] was performed based on the WHO criteria: FPG ≥ 7 mmol/L alone or 2 h after 75 g load glucose ≥ 11.1 mmol/L for T2DM, FPG 5.6–6.9 mmol/L or 2 h after 75 g load glucose 7.8–11.0 mmol/L for prediabetes. Bengtson et al. [48••] diagnosed impaired glucose tolerance as HbA1c ≥ 5.7%. Kondo et al. [24] diagnosed glucose intolerance with 75-g oral glucose tolerance tests. Among ten studies that reported T2DM as a primary outcome, greater than half used ADA criteria for diagnosis [60] (Table S2).

**Table 2:** Prediction temporality and sample size with respective events in included models

Articles	Country	Setting	Data source	Study design	Baseline time(t0)	Number of participants included	Number of events and percent	GI test time (t1)	Primary outcome
Bengtson 2022	USA	Clinical (hospital-based)	Questionnaires	Prospective cohort	2 days PP	203	71 (35%)	4–12 weeks	IGT
Man 2021	USA	Both	Secondary data	RCT	History	317	82 (25.9%)	3 years	DM
Bartáková 2021	Czech Republic	Clinical (hospital-based)	EHRs and questionnaires	Retrospective cohort (cross-sectional)	GDM Dx	244	22 (9%)	6–12 weeks	GI
Joglekar 2020	Australia	Clinical (hospital-based)	Questionnaires	Prospective cohort	12 Weeks pp	103	21 (20.4%)	(10 years)	T2DM
Muche, 2020	Ethiopia	Clinical (hospital-based)	MR and questionnaires	Prospective cohort study	GDM Dx	112	24 (21.4%)	6–12 weeks	GI
Khan 2019	Germany	Clinical (hospital-based)	EHRs	Nested case control study	6–9 Weeks pp	140	55(39.3%)	2 years	DM
Kondo 2018	Japan	Clinical (hospital-based)	MR	Retrospective cohort study	GDM Dx	123	45 (36.6%)	8–12 weeks	GI
Allalou 2016	USA	Clinical (hospital-based)	MR and questionnaires	Prospective cohort	6–9 Weeks pp	1010	122 (12.08%)	4 years	T2DM
Ignell 2016	Sweden	Clinical (hospital-based)	EHRs	A prospective cohort study	GDM Dx	362	72 (19%)	5 years	DM
Köhler 2016	Germany	Clinical (hospital-based)	Questionnaires	A prospective cohort study	GDM Dx	257	110 (42.8%)	20 years	DM
Bartáková 2015	Czech Republic	Clinical (hospital-based)	EHRs	Retrospective cohort	GDM Dx	305	51 (16.7%)	1 year	GA
Lappas 2015	Australia	Clinical (hospital-based)	MR and questionnaires	Prospective cohort	12 Month pp	104	21 (20.2%)	8–10 years	T2DM
Cormier 2015	Canada	Community based	Survey	A cohort study	PP	214	135 (63.08%)	3 years	pDM & DM
Kwak 2012	South Korea	Clinical (hospital-based)	MR	Prospective cohort study	GDM Dx	395	116 (29.4%)	45 months	T2DM
Kjos SL 1995	USA	Clinical (hospital-based)	MR and questionnaires	Prospective cohort	4–16 Weeks	671	146 (21.7%)	5–7 years	NIDDM

*EHRs* electronic health records, *MR* medical records, *GDM Dx* gestational diabetes mellitus diagnosis, *PP* postpartum, *T2DM* type 2 diabetes mellitus, *GI* glucose intolerance, *NIDDM* non-insulin dependent diabetes mellitus, *GA* glucose abnormality, *pDM* primary diabetes mellitus, *DM* diabetes mellitus, *IGT* impaired glucose intolerance, *USA* United States of America, *RCT* randomized controlled trial

### Predictors in the Final Model

**Fig. 2:**
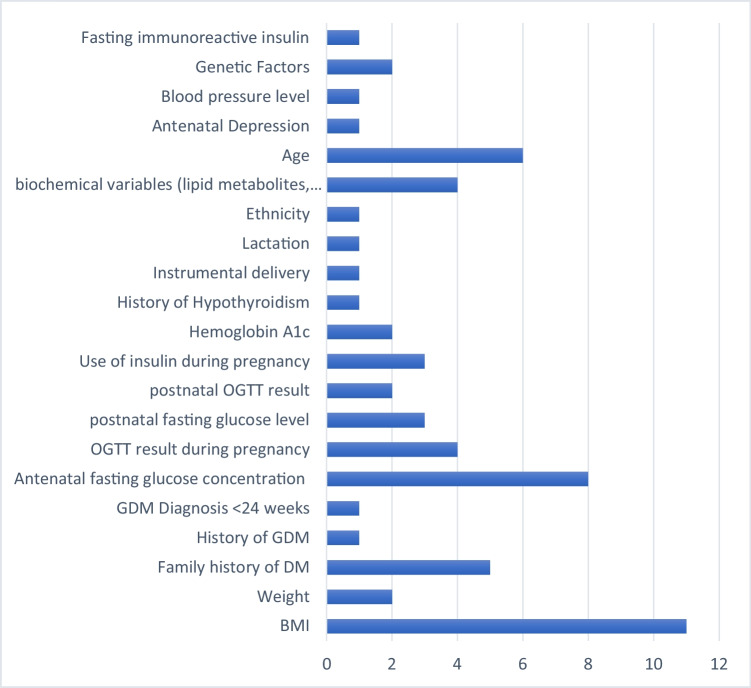
Predictors commonly utilized for developing prediction models to predict postpartum glucose intolerance in studies included in this systematic review

A list of predictors included in the final model is presented in Table 3. The number of risk predictor variables included in the models ranged from three to seven. Age (*n* = 6), FPG level during pregnancy (*n* = 8), and BMI (*n* = 11) were the three most common predictor variables included in the final model to predict postpartum glucose intolerance. Four models included biochemical variables such as branched-chain amino acids (BCAAs) (Val, Leu, Ile), lipid metabolites (sphingomyelin (SM (OH) C14:1), cholesteryl palmitic acid (CE(16:0)), non-esterified fatty acids (NEFA(22:4)), triglycerides and their fatty acid combination (TAG 48:2 FA 16:1, TAG 54:0 FA 16:0, TAG 50:1 FA 16:0), cholesteryl icosatetraenoate (CE(20:4)), phosphatidylethanolamine (PE(P-18:0/18:1), PE(P-36:2)), phosphatidylcholine (PC ae C40:5), hexoses, and phosphatidylserine (PS 38:4) [35–37, 55••]. Five models included a family history of diabetes mellitus, and four models included a 2-h plasma glucose level during pregnancy. Postnatal fasting glucose level, postnatal 2-h plasma glucose level, insulin therapy during pregnancy, and genetic factors were also other common prognostic determinants considered for model building. GDM history in a prior pregnancy, GDM diagnosis at < 24 weeks gestation, personal history of hypothyroidism, instrumental delivery, lactation, ethnicity, antenatal depression, blood pressure, genetic risk factors, and insulinogenic index/fasting immunoreactive insulin were each included only in one model (Fig. 2).

**Table 3:** Prognostic determinants included in the final model and their respective predictive performance

Study	Prognostic factors included in the final model	Discrimination		Calibration			Classification	
		C-statistics	AUC	Slope	Plot	HosLTest	Sensitivity	specificity
Bengtson 2022	Weight, BMI, family history of type 2 diabetes, GDM in a prior pregnancy, GDM diagnosis < 24 weeks’ gestation, and fasting and 2-h plasma glucose at 2 days postpartum		0.79 (0.72, 0.85)				80.0%	58.0%
Man 2021	Insulin treatment during pregnancy, BMI, fasting glucose level, HbA1c		0.74					
Bartáková 2021	FPG in mid-trimester OGTT above 5.1 mmol/L, obesity, family history of diabetes, instrumental delivery, personal history of hypothyroidism		0.83				82.4%	90.3%
Joglekar 2020	Age, BMI, pregnancy fasting glucose, postpartum fasting glucose, cholesterol, and triacylglycerols, circulating miR-369-3p measured at 12 weeks postpartum		0.92 (0.84, 1.00)				91.0%	89.0%
Muche 2020	Advanced maternal age, overweight and/or obesity, high FPG at GDM diagnosis, and antenatal depression		0.88 (0.82–0.94)			*P* = 0.759		
Khan 2019	Seven lipid metabolites (CE (16:0), NEFA (22:4), TAG 48:2 FA 16:1, CE (20:4), PE(P-18:0/18:1), CE (16:0), TAG 54:0 FA 16:0, TAG 50:1 FA 16:0)		0.92 (0.89,0.95)				87.0%	93.0%
Kondo 2018	Basic model (i.e. age, family history of diabetes, BMI > = 25 kg/m2, and use of insulin during pregnancy) plus insulinogenic index/fasting immunoreactive insulin < 1.1		0.71 (0.59–0.83)			P = 0.2102		
Allalou 2016	PC ae C40:5, hexoses, branched-chain amino acids (BCAAs) (Val, Leu, Ile), and SM (OH) C14:1		0.83 (0.76–0.89)				86.3%	69.0%
Ignell 2016	Ethnic origin, 2-h glucose concentrations during pregnancy, BMI		0.91		86.0%		82.1%	88.0%
Köhler 2016	Insulin treatment during pregnancy, family history of diabetes, BMI in early pregnancy, and lactation	0.76		1.13				
Bartáková 2015	HbA1c, FPG, 1-h post 75 g load glucose*							
Lappas 2015	Three lipids (CE 20:4, PE(P-36:2), and PS 38:4), six risk factors (age, BMI, and levels of pregnancy fasting glucose, postnatal fasting glucose, triacylglycerol, and total cholesterol)		0.86 (0.86, 0.87)				59.0%	89.9%
Cormier 2015	Age, BMI, explained-variance GRS		0.67 (0.60–0.73)					
Kwak 2012	Age, pre-pregnancy BMI, family history of diabetes, blood pressure, fasting glucose and fasting insulin concentration, wGRS	0.77						
Kjos SL 1995	The area under OGTT glucose curve at 4–14 weeks postpartum, Gestational age at diagnosis of GDM, the area under OGTT glucose curve during pregnancy, and the highest fasting serum glucose centration during pregnancy*							

*HosLTest* Hosmer–Lemeshow test, *BMI* body mass index, *GDM* gestational diabetes mellitus, *FPG* fasting plasma glucose, *oGTT* oral glucose tolerance test, *CE(16:0)* cholesteryl ester (16:0), *NEFA(22:4)* non-esterified fatty acids (22:4), *TAG 48:2 FA 16:1* triacylglycerol 48:2, fatty acid 16:1, *CE(20:4)* cholesteryl ester (16:0), *PE(P-18:0/18:1)* phosphatidylethanolamine, *TAG 54:0 FA 16:0* triacylglycerol 54:0, fatty acid 16:0, *TAG 50:1 FA 16:0* triacylglycerol 50:1, fatty acid 16:0, *HbA1c* hemoglobin A1c, *wGRS* weighted genetic risk score, *PC ae C40:5* phosphatidylcholine acyl-alkyl C40:5, *SM (OH) C14:1* C14:1-OH sphingomyelin

^*^The predictive performance is not reported

### Predictive Performance

Traditional statistical models were common, with only three applying machine learning (Table 4). The predictive performance of each study model is summarized in Table 3. The predictive performance of 13 studies that reported the area under the curve ranged from 0.66 to 0.92. However, none were externally validated. Only a few models were validated internally [35–37, 49••, 51, 55••]. Calibration was reported only for some models using Hosmer–Lemeshow test, calibration plot, and calibration slope [24, 51, 53].

**Table 4:** Data analysis method and modes of model presentation of the studies

Study	Missing data described	Missingness handling described	Statistical/data analysis methods used for model development	Mode of model presentation
Bengtson 2022	Yes	Yes	Lasso regression	NR
Man 2021	No	No	Multivariable Cox proportional hazards regression	Risk prediction equation
Bartáková 2021	No	No	Univariate and multivariate logistic regression with backward stepwise prediction algorithm	Risk score
Joglekar 2020	Yes	Yes	Univariate and multivariate logistic models were constructed to determine an eventual statistically significant effect of any relevant variable and ROC analysis was applied to test the final models. Machine learning and traditional analysis	NR
Muche 2020	No	No	Multivariable logistic regression	Risk prediction equation
Khan 2019	No	No	Stepwise multiple (both ways) logistic analysis and machine learning approach	Decision tree
Kondo 2018	No	No	Multivariable logistic regression analysis, decision-curve analysis	NR
Allalou 2016	No	No	Machine learning	Decision tree
Ignell 2016	Yes	No	Multivariable regression analysis	Model equation
Köhler 2016	No	No	Lasso method for Cox regression	Risk score
Bartáková 2015	No	No	Logistic regression analysis and ROC analysis	NR
Lappas 2015	Yes	No	Student’s *t* test, multivariate logistic regression analysis	NR
Cormier 2015	No	No	General linear model procedure and using the type-III sum of squares and logistic regression and ROC analysis	NR
Kwak 2012	Yes	Yes	Multiple logistic regression analysis	NR
Kjos SL1995	No	No	Multivariate regression analysis	NR

*NR* not reported, *ROC* receiver operating characteristic

### Risk of Bias Assessment and Meta-analysis

The risk of bias and applicability assessment results are shown in Table S1. Overall low risk of bias was present in two (2/15) studies only. Three domains including participant selection, predictor assessment, and outcome assessment resulted in a low risk of bias for most studies. A high risk of bias for participant selection was mainly due to controversial inclusion or exclusion criteria Table S3. In addition, two studies selected participants for inclusion based on one question only asking “Have you ever been told that you had a high sugar level during pregnancy?” which is a less sensitive approach, potentially introducing serious bias and may compromise the transportability of the model [49••, 57]. The answer to the question “Were predictor assessments made without knowledge of outcome data?” resulted in a high risk of bias because the assessment of predictors was performed in retrospect after the outcome was known and/or there was no statement showing whether assessors were blinded or not. As most models had less than 10 events per variable (EPV) or the EPV was unable to be extracted (Table 2), this was scored as a high risk of bias for most models.

However, in the analysis section, most studies had a high risk of bias. In some studies, continuous and categorical variables were not handled appropriately. For instance, although using continuous variable is recommended in prediction model development, Man (2021) categorized age into six categories and BMI into three categories. The analysis did not include all enrolled participants and/or did not report on those who were excluded. Additionally, participants with missing data were not adequately addressed and/or were not reported. For example, Ignell (2016), which relies on prospective data collection, experienced a significant amount of lost to follow-up. The selection of predictors based on univariable analysis was applied and/or not reported [48••, 58••], and complexities in the data were not accounted for appropriately and/or not reported. Relevant model performance measures were not evaluated appropriately and/or not reported. Furthermore, model overfitting and optimism in model performance were not accounted for and/or not reported. Predictors and their assigned weights in the final model did not correspond to the results from the reported multivariable analysis and/or are not reported.

Meta-analysis was not possible due to the lack of homogeneity in methods used to develop the prediction models, different sets of predictors used to develop different prediction models, and heterogeneity in the prediction time interval ranging from 1 to 20 years postpartum.

## Discussion

This systematic review of risk models predicting postpartum glucose intolerance among women who had GDM identified 15 models; however, none were externally validated and less than half were internally validated. No models had the same set of prognostic factors, and factors included a range of demographic, clinical, and biomarker factors. The most frequent factors were BMI (measured pre-pregnancy or early pregnancy), fasting glucose concentration during pregnancy, maternal age, and family history of T2DM. Some studies included only traditional clinical risk factors (e.g., age, BMI, pregnancy fasting OGTT, and postnatal fasting OGTT), while others included biochemical variables and genetic factors. Among traditional risk factors, the most common potentially modifiable factor was BMI (pre-pregnancy/early pregnancy). Predictive performances were suggested to be above chance (with AUC > 0.66); however, performance was difficult to evaluate as all included studies had a high risk of bias with various methodological shortcomings.

The type of prognostic factors used in the models depended on the time when risk for postpartum glucose intolerance was assessed. Some studies used clinical and biochemical factors collected during and/or before GDM diagnosis, thus making the GDM diagnosis the starting point (baseline) for the prediction. However, other studies had baseline risk assessments after delivery at 2 days [48••], 6–9 weeks [35, 36], 12 weeks [55••], 4–16 weeks [50], and 12 months [37]. In these latter studies, additional prognostic factors included postnatal fasting and 2-h plasma glucose [37, 51, 55••], mode of delivery [52••], lactation [51], and circulating miR-369-3p measured at 12 weeks postpartum [55••]. Future studies are warranted to examine which baseline time point and prognostic factors are associated with the most accurate prediction.

This review has highlighted that, although study participants were defined as women with GDM, the inclusion criteria applied were not always rigorous; for example, two studies selected participants based on one question only asking “Have you ever been told that you had a high sugar level during pregnancy?” 49••, 57. Instead, wherever possible, a diagnostic test or robust selection criteria should be applied to distinguish the target population to be included in the study [61, 62]. Otherwise, ambiguous population groups can lead to excess variability in the study data, making prediction difficult, therefore limiting any usefulness of the models and eventual inclusion in subsequent meta-analyses.

Many of these studies used routinely collected health data which is more generalizable at population level. However, missing data are not uncommon when examining routinely collected health data and retrospective cohort studies which may reduce the available evidence to build the model [63]. Instead of ignoring variables having missing data, which can introduce a source of serious bias, it is suggested that missing data should be replaced based on the available information by using advanced methods such as multiple imputations [64]. However, only a few models discussed missing data [48••, 53, 55••, 56], and only Bengtson et al. [48••] and Kwak et al. [56] applied multiple imputations to handle missing data. Where other studies instead excluded participants due to missing clinical data, this may aggravate the problem of small sample size and discards the information of nearly complete data [55••].

Comparing the predictive performance of the included studies is not a straightforward task, as the predictors utilized for each model’s development vary. Nonetheless, it can be observed that, on average, machine learning algorithms outperform traditional models in terms of sensitivity and specificity. This can be attributed to their capacity to identify complex patterns and relationships in the data that may not be apparent to the naked eye. It is worth noting, however, that traditional models may have advantages in terms of being more interpretable and simpler. Although some models show performance measures suggesting excellent predictive capabilities, our review found that none were externally validated and only a few were internally validated. This lack of validation puts their reproducibility under question. Therefore, testing the model performance in a new population in a different geographic region or in different time period is required to further this field and to assess the practical utility of the model.

Women who have experienced GDM are 8 to 10 times and 2 times at higher risk of developing type 2 diabetes and cardiovascular disease (CVD), respectively [5, 65–68]. The above emphasizes the pressing requirement for timely and continuous proactive monitoring, as well as efficient preventative measures, for type 2 diabetes and cardiovascular disease (CVD). Among these strategies, developing a well-designed clinical prediction model based on historical, antepartum, and even early postpartum variables is mandatory to early identification of at-risk women and early initiation of intervention. To be more precise, the screening and prevention of T2DM related to gestational diabetes is a subject that is challenging and controversial [69] and would benefit greatly from the development of a thoroughly planned and validated prediction model.

### Strengths and Limitations

This is the first systematic review of risk prediction studies of postpartum glucose intolerance among women who have a history of GDM. Strengths of this review are that the search strategy was built based on a validated search strategy for prediction models, and the quality of risk prediction models was assessed by CHARMS guidelines. Limitations for deriving information from this review mostly arise from the low quality of the identified eligible studies. However, examining the overall quality and characteristics of existing models is important to understand the flaws and strengths of developed models, using these as stepping stones to build novel models in future.

A major limitation of the studies identified is that very few followed the reporting guidelines for prognostic risk prediction modeling. Researchers examining this area are strongly recommended to follow the appropriate guidelines so that this area can be advanced. Another major limitation of the models identified was that there was a high risk of bias evident in all included studies. The various methodological shortcomings included the use of inadequate sample sizes, uncertain inclusion or exclusion criteria, lack of missing data reporting and/or handling, inappropriate management of continuous and categorical variables, use of univariable analysis for selection of predictors, failing to evaluate/report relevant model performance measures, failing to consider model overfitting and optimism in model performance, lack of internal and external validation, the low trend of model performance measure reporting, and lack of model presentation.

Furthermore, only a fraction of models considered overfitting. Overfitting is especially prevalent when there are too few outcome events as compared with candidate prognostic determinants. Additionally, overfitting is expected when the model is developed in a small dataset, inappropriate continuous variables categorization is employed, and when stepwise predictor selection methods based on significance criteria are applied [70, 71]. In the included studies, some had very small sample sizes (*n* = 103, event = 21) [55••], (*n* = 104, event = 21) [37], (*n* = 112, event = 24) [58••], (*n* = 123, event = 45) [24] (*n* = 140, event = 55) [36], (*n* = 203, event = 71) [48••]. If the number of predictors considered for prediction is larger than the number of events of interest, the predictive performance will be overestimated. Preferably, predictive model studies necessitate a minimum of several hundred outcome events [72]. Small samples and a reduced number of events compared to several predictors will lead to overfitting and compromise the transportability of the model in a similar or a different population. This is important especially in regions with increasing migration, and the propensity for some groups to “adopt” a higher risk in the new home; therefore, external validation and ultimately generalisable models are needed more than ever.

## Conclusions

GDM is common, and rates are rising globally. Women with this condition have a high risk of conversion to glucose intolerance postpartum. Identification of those at risk can facilitate targeted screening and prevention strategies. Despite this, our systematic review identified that existing prognostic models for glucose intolerance following GDM were not externally validated, and only a few were internally validated. In addition, there was a high risk of bias, unreported model calibration, and low use of model presentation methods. Future research should focus on the development of robust, high-quality risk prediction models by incorporating easily accessible prognostic determinates to enhance the practical application and accuracy of risk prediction models for glucose intolerance and T2DM following GDM looking the summarized result of this review. External validation is also required before implementing these prediction models into clinical practice.

## Supplementary Information

Below is the link to the electronic supplementary material.

Supplementary file1 (DOCX 18 KB)

Supplementary file2 (DOCX 17 KB)

Supplementary file3 (DOCX 16 KB)

Supplementary file4 (DOCX 29 KB)

Supplementary file5 (DOCX 17 KB)

## Data Availability

Data are available upon request from the corresponding author.

## References

[1] Steegers EAP, Fauser BCJM, Hilders CGJM, Jaddoe VWV, Massuger LFAG, van der Post JAM et al. Textbook of obstetrics and gynaecology: a life course approach. Bohn Stafleu Van Loghum. 2019. 10.1007/978-90-368-2131-5.

[2] Wang H, Li N, Chivese T, Werfalli M, Sun H, Yuen L (2022). IDF diabetes atlas: estimation of global and regional gestational diabetes mellitus prevalence for 2021 by international association of diabetes in pregnancy study group’s criteria. Diabetes Res Clin Pract..

[3] McIntyre HD, Catalano P, Zhang C, Desoye G, Mathiesen ER, Damm P (2019). Gestational diabetes mellitus. Nat Rev Dis Primers.

[4] Yogev Y, Visser GH (2009). Obesity, gestational diabetes and pregnancy outcome. Semin Fetal Neonatal Med.

[5] Song C, Lyu Y, Li C, Liu P, Li J, Ma RC (2018). Long-term risk of diabetes in women at varying durations after gestational diabetes: a systematic review and meta-analysis with more than 2 million women. Obes Rev.

[6] Zhu Y, Zhang C (2016). Prevalence of gestational diabetes and risk of progression to type 2 diabetes: a global perspective. Curr Diab Rep.

[7] Catalano PM, McIntyre HD, Cruickshank JK, McCance DR, Dyer AR, Metzger BE (2012). The hyperglycemia and adverse pregnancy outcome study: associations of GDM and obesity with pregnancy outcomes. Diabetes Care.

[8] Bennett CJ, Walker RE, Blumfield ML, Gwini SM, Ma J, Wang F (2018). Interventions designed to reduce excessive gestational weight gain can reduce the incidence of gestational diabetes mellitus: a systematic review and meta-analysis of randomised controlled trials. Diabetes Res Clin Pract.

[9] Rahati S, Shahraki M, Arjomand G, Shahraki T (2014). Food pattern, lifestyle and diabetes mellitus. Int J High Risk Behav Addict..

[10] Moyer VA, U.S. Preventive Services Task Force (2014). Screening for gestational diabetes mellitus: U.S. Preventive Services Task Force recommendation statement. Ann Intern Med..

[11] Kim C, Newton KM, Knopp RH (2002). Gestational diabetes and the incidence of type 2 diabetes: a systematic review. Diabetes Care.

[12] Leuridan L, Wens J, Devlieger R, Verhaeghe J, Mathieu C, Benhalima K (2015). Glucose intolerance in early postpartum in women with gestational diabetes: who is at increased risk?. Prim Care Diabetes.

[13] Kim SH, Kim MY, Yang JH, Park SY, Yim CH, Han KO (2011). Nutritional risk factors of early development of postpartum prediabetes and diabetes in women with gestational diabetes mellitus. Nutrition.

[14] Fatin AAB, Alina TI (2019). Proportion of women with history of gestational diabetes mellitus who performed an oral glucose test at six weeks postpartum in Johor Bahru with abnormal glucose tolerance. Malays Fam Physician..

[15] England LJ, Dietz PM, Njoroge T, Callaghan WM, Bruce C, Buus RM (2009). Preventing type 2 diabetes: public health implications for women with a history of gestational diabetes mellitus. Am J Obstet Gynecol.

[16] Bellamy L, Casas JP, Hingorani AD, Williams D (2009). Type 2 diabetes mellitus after gestational diabetes: a systematic review and meta-analysis. Lancet.

[17] ACOG Practice Bulletin No. 190 (2018). ACOG Practice Bulletin No. 190 Summary: gestational diabetes mellitus. Obstet Gynecol..

[18] American Diabetes Association. 14 (2020). Management of diabetes in pregnancy: standards of medical care in Diabetes-2020. Diabetes Care..

[19] Kwak SH, Choi SH, Jung HS, Cho YM, Lim S, Cho NH (2013). Clinical and genetic risk factors for type 2 diabetes at early or late post partum after gestational diabetes mellitus. J Clin Endocrinol Metab.

[20] Jang HC (2011). Gestational diabetes in Korea: incidence and risk factors of diabetes in women with previous gestational diabetes. Diabetes Metab J.

[21] Buchanan TA, Xiang A, Kjos SL, Lee WP, Trigo E, Nader I (1998). Gestational diabetes: antepartum characteristics that predict postpartum glucose intolerance and type 2 diabetes in Latino women. Diabetes.

[22] Metzger BE, Cho NH, Roston SM, Radvany R (1993). Prepregnancy weight and antepartum insulin secretion predict glucose tolerance five years after gestational diabetes mellitus. Diabetes Care.

[23] Feig DS, Zinman B, Wang X, Hux JE (2008). Risk of development of diabetes mellitus after diagnosis of gestational diabetes. CMAJ.

[24] Kondo M, Nagao Y, Mahbub MH, Tanabe T, Tanizawa Y (2018). Factors predicting early postpartum glucose intolerance in Japanese women with gestational diabetes mellitus: decision-curve analysis. Diabet Med.

[25] Kugishima Y, Yasuhi I, Yamashita H, Fukuda M, Kuzume A, Sugimi S (2015). Risk factors associated with abnormal glucose tolerance in the early postpartum period among Japanese women with gestational diabetes. Int J Gynaecol Obstet.

[26] Kojima N, Tanimura K, Deguchi M, Morizane M, Hirota Y, Ogawa W (2016). Risk factors for postpartum glucose intolerance in women with gestational diabetes mellitus. Gynecol Endocrinol.

[27] Masuko N, Tanimura K, Kojima N, Imafuku H, Deguchi M, Okada Y (2022). Predictive factors for postpartum glucose intolerance in women with gestational diabetes mellitus. J Obstet Gynaecol Res.

[28] Saisho Y, Miyakoshi K, Tanaka M, Matsumoto T, Minegishi K, Yoshimura Y (2012). Antepartum oral disposition index as a predictor of glucose intolerance postpartum. Diabetes Care..

[29] Dupuis J, Langenberg C, Prokopenko I, Saxena R, Soranzo N, Jackson AU (2010). New genetic loci implicated in fasting glucose homeostasis and their impact on type 2 diabetes risk. Nat Genet.

[30] Voight BF, Scott LJ, Steinthorsdottir V, Morris AP, Dina C, Welch RP (2010). Twelve type 2 diabetes susceptibility loci identified through large-scale association analysis. Nat Genet.

[31] Cho YS, Chen CH, Hu C, Long J, Ong RTH, Sim X (2011). Meta-analysis of genome-wide association studies identifies eight new loci for type 2 diabetes in east Asians. Nat Genet.

[32] Morris AP, Voight BF, Teslovich TM, Ferreira T, Segrè AV, Steinthorsdottir V (2012). Large-scale association analysis provides insights into the genetic architecture and pathophysiology of type 2 diabetes. Nat Genet.

[33] Wu NN, Zhao D, Ma W, Lang JN, Liu SM, Fu Y (2021). A genome-wide association study of gestational diabetes mellitus in Chinese women. J Matern Fetal Neonatal Med.

[34] Wang XM, Gao Y, Eriksson JG, Chen W, Chong YS, Tan KH (2021). Metabolic signatures in the conversion from gestational diabetes mellitus to postpartum abnormal glucose metabolism: a pilot study in Asian women. Sci Rep.

[35] Allalou A, Nalla A, Prentice KJ, Liu Y, Zhang M, Dai FF (2016). A predictive metabolic signature for the transition from gestational diabetes mellitus to type 2 diabetes. Diabetes.

[36] Khan SR, Mohan H, Liu Y, Batchuluun B, Gohil H, Al Rijjal D (2019). The discovery of novel predictive biomarkers and early-stage pathophysiology for the transition from gestational diabetes to type 2 diabetes. Diabetologia.

[37] Lappas M, Mundra PA, Wong G, Huynh K, Jinks D, Georgiou HM (2015). The prediction of type 2 diabetes in women with previous gestational diabetes mellitus using lipidomics. Diabetologia.

[38] Tobias DK, Clish C, Mora S, Li J, Liang L, Hu FB (2018). Dietary intakes and circulating concentrations of branched-chain amino acids in relation to incident type 2 diabetes risk among high-risk women with a history of gestational diabetes mellitus. Clin Chem.

[39] Brown SD, Hedderson MM, Zhu Y, Tsai AL, Feng J, Quesenberry CP (2022). Uptake of guideline-recommended postpartum diabetes screening among diverse women with gestational diabetes: associations with patient factors in an integrated health system in the USA. BMJ Open Diabetes Res Care..

[40] Linnenkamp U, Greiner GG, Haastert B, Adamczewski H, Kaltheuner M, Weber D (2022). Postpartum screening of women with GDM in specialised practices: data from 12,991 women in the GestDiab register. Diabet Med..

[41] Hu FB, Manson JE, Stampfer MJ, Colditz G, Liu S, Solomon CG (2001). Diet, lifestyle, and the risk of type 2 diabetes mellitus in women. N Engl J Med.

[42] Zhang Y, Pan XF, Chen J, Xia L, Cao A, Zhang Y (2020). Combined lifestyle factors and risk of incident type 2 diabetes and prognosis among individuals with type 2 diabetes: a systematic review and meta-analysis of prospective cohort studies. Diabetologia.

[43] Page MJ, Moher D, Bossuyt PM, Boutron I, Hoffmann TC, Mulrow CD (2021). PRISMA 2020 explanation and elaboration: updated guidance and exemplars for reporting systematic reviews. BMJ..

[44] Moons KGM, de Groot JAH, Bouwmeester W, Vergouwe Y, Mallett S, Altman DG (2014). Critical appraisal and data extraction for systematic reviews of prediction modelling studies: the CHARMS checklist. PLOS Med..

[45] World Health Organization. HEARTS D: diagnosis and management of type 2 diabetes. World Health Organization. 2020. https://apps.who.int/iris/handle/10665/331710. License: CC BY-NC-SA 3.0 IGO.

[46] Zidek JV, Wong H, Le ND, Burnett R (1996). Causality, measurement error and multicollinearity in epidemiology. Environmetrics.

[47] Yoo W, Mayberry R, Bae S, Singh K, Peter He Q, Lillard JW (2014). A study of effects of multicollinearity in the multivariable analysis. Int J Appl Sci Technol..

[48] •• Bengtson AM, Dice ALE, Clark MA, Gutman R, Rouse D, Werner E. Predicting progression from gestational diabetes to impaired glucose tolerance using peridelivery data: an observational Study. Am J Perinatol. 2022. 10.1055/a-1877-9587. **A prediction model was created to forecast the risk of postpartum glucose intolerance in women with gestational diabetes mellitus (GDM) using prognostic factors that are easily accessible**. 10.1055/a-1877-9587 35709723

[49] •• Man B, Schwartz A, Pugach O, Xia Y, Gerber B. A clinical diabetes risk prediction model for prediabetic women with prior gestational diabetes. Plos One. 2021;16(6):e0252501. 10.1371/journal.pone.0252501. **A prediction model was created to identify the risk of postpartum prediabetes in women with gestational diabetes mellitus (GDM) at an early stage. The model utilizes four prognostic factors to make the prediction**. 10.1371/journal.pone.0252501 PMC8232404 34170930

[50] Kjos SL, Peters RK, Xiang A, Henry OA, Montoro M, Buchanan TA (1995). Predicting future diabetes in Latino women with gestational diabetes. Utility of early postpartum glucose tolerance testing. Diabetes..

[51] Köhler M, Ziegler AG, Beyerlein A (2016). Development of a simple tool to predict the risk of postpartum diabetes in women with gestational diabetes mellitus. Acta Diabetol.

[52] Bartáková V, Barátová B, Pácal L, Ťápalová V, Šebestová S, Janků P (2021). Development of a new risk score for stratification of women with gestational diabetes mellitus at high risk of persisting postpartum glucose intolerance using routinely assessed parameters. Life (Basel).

[53] Ignell C, Ekelund M, Anderberg E, Berntorp K (2016). Model for individual prediction of diabetes up to 5 years after gestational diabetes mellitus. Springerplus.

[54] Bartáková V, Malúšková D, Mužík J, Bělobrádková J, Kaňková K (2015). Possibility to predict early postpartum glucose abnormality following gestational diabetes mellitus based on the results of routine mid-gestational screening. Biochem Med.

[55] Joglekar MV, Wong WKM, Ema FK, Georgiou HM, Shub A, Hardikar AA (2021). Postpartum circulating microRNA enhances prediction of future type 2 diabetes in women with previous gestational diabetes. Diabetologia.

[56] Kwak SH, Choi SH, Kim K, Jung HS, Cho YM, Lim S (2013). Prediction of type 2 diabetes in women with a history of gestational diabetes using a genetic risk score. Diabetologia.

[57] Cormier H, Vigneault J, Garneau V, Tchernof A, Vohl MC, Weisnagel SJ (2015). An explained variance-based genetic risk score associated with gestational diabetes antecedent and with progression to pre-diabetes and type 2 diabetes: a cohort study. BJOG.

[58] •• Muche AA, Olayemi OO, Gete YK. Predictors of postpartum glucose intolerance in women with gestational diabetes mellitus: a prospective cohort study in Ethiopia based on the updated diagnostic criteria. BMJ Open. 2020;10(8):e036882. 10.1136/bmjopen-2020-036882. **Developed a prediction model for predicting risk of postpartum glucose intolerance among women with GDM by using the following factors: advanced maternal age, overweight and/or obesity, high FPG at GDM diagnosis, and antenatal depression**. 10.1136/bmjopen-2020-036882 PMC7462231 32868358

[59] World Health Organization & International Diabetes Federation. Definition and diagnosis of diabetes mellitus and intermediate hyperglycaemia: report of a WHO/IDF consultation. World Health Organization. 2006. https://apps.who.int/iris/handle/10665/43588.

[60] American Diabetes Association. Research, Education, Advocacy [cited Oct 6 2022]. Available from: https://diabetes.org/.

[61] Grobbee DE, Hoes AW. Clinical epidemiology: principles, methods, and applications for clinical research. 2nd ed. Jones & Bartlett Learning; 2014.

[62] Moons KGM, Altman DG, Reitsma JB, Ioannidis JPA, Macaskill P, Steyerberg EW (2015). Transparent Reporting of a multivariable prediction model for Individual Prognosis or Diagnosis (TRIPOD): explanation and elaboration. Ann Intern Med.

[63] Clark TG, Altman DG (2003). Developing a prognostic model in the presence of missing data: an ovarian cancer case study. J Clin Epidemiol.

[64] Royston P, Moons KGM, Altman DG, Vergouwe Y (2009). Prognosis and prognostic research: developing a prognostic model. BMJ..

[65] Vounzoulaki E, Khunti K, Abner SC, Tan BK, Davies MJ, Gillies CL (2020). Progression to type 2 diabetes in women with a known history of gestational diabetes: systematic review and meta-analysis. BMJ..

[66] Li Z, Cheng Y, Wang D, Chen H, Chen H, Ming WK, Wang Z (2020). Incidence rate of type 2 diabetes mellitus after gestational diabetes mellitus: a systematic review and meta-analysis of 170,139 women. J Diabetes Res.

[67] Nielsen KK, Kapur A, Damm P, de Courten M, Bygbjerg IC (2014). From screening to postpartum follow-up – the determinants and barriers for gestational diabetes mellitus (GDM) services, a systematic review. BMC Pregnancy Childbirth.

[68] Kramer CK, Campbell S, Retnakaran R (2019). Gestational diabetes and the risk of cardiovascular disease in women: a systematic review and meta-analysis. Diabetologia.

[69] Balaji B, Ranjit Mohan AR, Rajendra P, Mohan D, Ram U, Viswanathan M (2019). Gestational diabetes mellitus postpartum follow-up testing: challenges and solutions. Can J Diabetes.

[70] Harrell FE. Regression modeling strategies: with applications to linear models, logistic regression, and survival analysis. New York: Springer; 2001. 10.1007/978-1-4757-3462-1

[71] Ohlssen D (2009). A review of: ”clinical prediction models: a practical approach to development, validation, and updating, by E.W. Steyerberg”. J Biopharm Stat..

[72] Moons KGM, Royston P, Vergouwe Y, Grobbee DE, Altman DG (2009). Prognosis and prognostic research: what, why, and how?. BMJ..

