# Supplementary material for: Models Predicting Postpartum Glucose Intolerance Among Women with a History of Gestational Diabetes Mellitus: a Systematic Review
Source: Curr Diab Rep. 2023 Jun 9;23(9):231–43. doi: 10.1007/s11892-023-01516-0 (PMC10435618; doi:10.1007/s11892-023-01516-0)
Supplement: Supplementary file 1 — Supplementary file1 (DOCX 18 KB) [file 11892_2023_1516_MOESM1_ESM.docx]

**Table S1. Risk of bias and applicability assessment result**

| **Study** | **Risk of bias** | | | | **Applicability** | | | **Overall** | |
| --- | --- | --- | --- | --- | --- | --- | --- | --- | --- |
|  | Participants | Predictors | Outcome | Analysis | Participants | Predictors | Outcome | ROB | Applicability |
| Bengtson 2022 | - | + | + | + | + | + | + | + | + |
| Man 2021 | - | + | + | - | + | - | + | - | - |
| Bartáková 2021 | + | - | + | - | + | + | + | - | + |
| Joglekar 2020 | - | + | + | + | + | + | + | + | + |
| Muche 2020 | + | + | + | - | + | + | + | - | + |
| Khan 2019 | + | + | + | - | + | + | + | - | + |
| Kondo 2018 | - | - | + | - | + | + | + | - | + |
| Allalou 2016 | + | + | + | - | + | + | + | - | + |
| Ignell 2016 | + | + | + | - | + | + | + | - | + |
| Köhler 2016 | + | + | + | - | + | + | + | - | + |
| Bartakova 2015 | + | - | + | - | + | + | + | - | + |
| Lappas 2015 | - | + | + | - | + | + | + | - | + |
| Cormier 2015 | - | + | + | - | + | + | + | - | + |
| Kwak 2012 | + | + | + | - | + | + | + | - | + |
| Kjos SL 1995 | + | + | + | - | + | + | + | - | + |

*ROB: Risk of Bias; + indicates low ROB/low concern regarding applicability; - indicates high ROB/high concern regarding applicability; and? indicates unclear ROB/unclear concern regarding applicability.*
