# Supplementary material for: Models Predicting Postpartum Glucose Intolerance Among Women with a History of Gestational Diabetes Mellitus: a Systematic Review
Source: Curr Diab Rep. 2023 Jun 9;23(9):231–43. doi: 10.1007/s11892-023-01516-0 (PMC10435618; doi:10.1007/s11892-023-01516-0)
Supplement: Supplementary file 2 — Supplementary file2 (DOCX 17 KB) [file 11892_2023_1516_MOESM2_ESM.docx]

**Table S2. Gestational diabetes mellitus and glucose intolerance diagnosis method and primary outcomes of the included models**

| **Study** | **GDM Diagnosis Method** | **Glucose Intolerance diagnosis method** |
| --- | --- | --- |
| **Bengtson 2022** | By a 1-hour glucose challenge test value > 200 mg/dl or by the Carpenter Coustan criteria for the 3-hour 100-gram OGTT | HbA1c > = 5.7%. |
| **Man 2021** | Have you ever been told that you had a high sugar level or that you have diabetes and selected the answer Only during pregnancy were considered to have had GDM | Diagnostic criteria for diabetes was defined by a fasting plasma glucose ≥ 140 mg/dL (until  June 23, 1997) or ≥ 126 mg/dL (on or after June 24, 1997), or a 2-hour 75-gram oral glucose  tolerance test (OGTT) ≥ 200 mg/dL |
| **Bartáková 2021** | GDM was diagnosed according to the **old WHO criteria**: fasting plasma glucose (FPG) 5.6 mmol/L, 1-h post-load glucose 8.9 mmol/L and 2-h post-load glucose 7.7 mmol/L (reaching any of the three cut-off values qualified for the GDM diagnosis | Post-partum diagnosis of diabetes/prediabetes was based on the WHO criteria for non-pregnant subjects: FPG ≥ 7 mmol/L alone or 2-h post-load glucose ≥ 11.1 mmol/L for diabetes mellitus, FPG 5.6–6.9 mmol/L or 2-h post-load glucose 7.8–11.0 mmol/L for prediabetes". In case of positive postpartum test for manifest diabetes urinary ketone bodies, C-peptide and selected antibodies (anti-glutamic acid decarboxylase, anti-tyrosine phosphataseâ€“2, and insulin autoantibodies) were measured to identify eventual type 1 diabetes (T1DM) oGTT |
| **Joglekar 2020** | OGTT at 24 -28 weeks of gestation, according to the Australasian Diabetes in Pregnancy Society (ADIPS) guidelines of the period, with a fasting venous plasma glucose concentration of >5.5 mmol/l and/or > 8.0 mmol/l glucose 2 h after a 75 g oral glucose load. | Participants were classified as having progressed to type 2 diabetes based on the ADA criteria |
| **Muche 2020** | The universal screening for GDM using a 2-hour 75g OGTT was performed for all pregnant women at 24 - 28 weeks by capillary glucose testing, using a standard plasma-calibrated glucometer. The updated diagnostic criteria for GDM diagnosis were made according to the 2017 American Diabetes Association, 2013 WHO, or modified International Association of Diabetes and Pregnancy Study Groups. The diagnosis of GDM is made when one or more of the values of plasma glucose level were met (fasting: > 92mg/dL; 1hour: > 180mg/dL; 2hours: > 153mg/dL). | The primary outcome was diagnosis of postpartum pre-diabetes (impaired fasting glucose (IFG): FPG 100 - 125mg/dL; impaired glucose tolerance (IGT): 2-hour plasma glucose in 75g OGTT 140 - 199mg/dL) or diabetes (FPG - 126mg/dL, or 2-hour plasma glucose > 200mg/dL in OGTT or random plasma glucose > 200mg/dL) |
| **Khan 2019** | GDM diagnosis is based on standardized 3-hour 100 g oral glucose tolerance tests during pregnancy using Carpenter and Coustan's criteria as recommended by the A**merican Diabetes Association (ADA)**. Two or more of the four plasma glucose values have to meet or exceed the plasma glucose thresholds recommended by the ADA and the American College of Obstetricians and Gynecologists (ACOG) and received standard treatment for GDM within by KPNC prenatal care providers. | The 2-hr 75-gram OGTT at 6-9 weeks postpartum as recommended by the ADA |
| **Kondo 2018** | Pregnant women with casual blood glucose levels of 5.6 mmol/l or those with 1-h glucose levels of 7.8 mmol/l on 50-g glucose challenge tests underwent a 75-g OGTT. All of the women had been diagnosed with GDM based on the International Association of Diabetes and Pregnancy Study Groups criteria | 75-g oral glucose tolerance tests. According to the WHO 1999 criteria, Type 2 diabetes was diagnosed when fasting blood glucose levels were > 7 mmol/l or 2-h blood glucose levels were > 11.1 mmol/l. Impaired fasting glucose was diagnosed when fasting blood glucose levels were > 6.1 mmol/l, and impaired glucose tolerance when 2-h blood glucose levels were > 7.8 mmol/l. Fasting blood glucose levels of<6.1 mmol/l and 2-h blood glucose levels of<7.8 mmol/l were identified as normal. |
| **Allalou 2016** | 3-h 100-g OGTT based on the Carpenter and Coustan criteria | T2D diagnosis was based on ADA criteria |
| **Ignell 2016** | The diagnostic criteria for GDM used in the present study were a modification of those recommended by the WHO in 1999, defining GDM as the joint category of diabetes and impaired glucose tolerance (IGT) based on the 2-h capillary plasma glucose concentration (World Health Organization 1999). A 2-h capillary plasma glucose concentration below the limit for IGT was considered to be normal glucose tolerance (NGT) during pregnancy (GNGT) (World Health Organization 199 | Fasting and 2-h venous blood samples were drawn in duplicate for determination of plasma glucose concentration, and the mean value was calculated. Diagnostic criteria were those proposed by the WHO (World Health Organization 1999) |
| **Köhler 2016** | According to the criteria of the **German Diabetes Association**, using an oral glucose tolerance test (OGTT) with 75-g glucose load. GDM was diagnosed if two out of three capillary blood glucose values exceeded the following limits: >5.0 mmol/1 (fasting) before OGTT, >10.6 mmol/1 after 60 min, and >8.9 mmol/1 after 120 min | OGTTs, diabetes during follow-up was diagnosed according to World Health Organization criteria, WHO 1990 |
| **Bartáková 2015** | based on the consensus criteria of Czech Diabetes Society and Czech Society for Clinical Biochemistry derived from WHO criteria for subjects with impaired fasting glucose (IFG) / impaired glucose tolerance (IGT) using a 3-point oGTT with 75 g glucose with threshold values (any value above cut-off diagnosing GDM): FPG 5.6 mmol/L, 1-hr after 75 g load glucose 8.8 mmol/L and/or 2-hr after 75 g load > 7.8mmol/L (sample 60 minutes after challenge optional but recommended | Postpartum diagnosis of diabetes/prediabetes was based on the WHO criteria: FPG > 7mmol/L alone or 2-hr after 75 g load glucose > 11.1mmol/L for DM, FPG 5.6 - 6.9mmol/L or 2-hr after 75 g load glucose 7.8-11.0 mmol/L for prediabetes.  In the case of postpartum diagnosis of manifest diabetes urinary ketone bodies, C-peptide and antibodies (anti-glutamic acid decarboxylase (anti-GAD), anti-tyrosine phosphatase (antiIA-2), insulin auto-anti-bodies (IAA)) were measured to identify an even-tual T1DM. |
| **Lappas 2015** | The diagnosis of GDM was made by an OGTT at 24 - 28 weeks of gestation, according to the Australasian Diabetes in Pregnancy Society (ADIPS) guidelines by either a fasting venous plasma concentration of > 5.5 mmol/l glucose and/or > 8.0 mmol/l glucose 2 h after a 75 g oral glucose load. | type 2diabetes according to ADA criteria |
| **Cormier 2015** | Have you ever been told that you had a high sugar level during pregnancy | In accordance with the 2013 Canadian Diabetes Association latest guidelines, pre-diabetes was defined as impaired fasting glucose, and/or impaired glucose tolerance, and/or A1C (between 6.0 and 6.4%). T2D was defined as fasting plasma glucose > 7.0 mmol/l, and/or 2-hour plasma glucose post-OGTT > 11.1 mmol/l, and/or A1C > 6.5% |
| **Kwak 2012** | The diagnosis of GDM was made if two or more of the following criteria were met during the 100 g OGTT, according to the criteria of the Third International Workshop-Conference on Gestational Diabetes Mellitus: fasting glucose >5.8 mmol/l, 1 h glucose >10.6 mmol/l, 2 h glucose >9.2 mmol/l, 3 h glucose >8.1 mmol/l. Insulin levels were also measured during the 100 g OGTT. | All women with GDM were tested 2 months postpartum and annually afterward with a 75 g OGTT at each visit. We classified participants as having normal glucose tolerance (NGT), impaired glucose tolerance (IGT), or type 2 diabetes according to American Diabetes Association criteria. |
| **Kjos SL1995** | According to National Diabetes Data Group (NDDG) recommendations | The diagnosis of diabetes was based on a fasting plasma glucose level >7.8 mmol/1 in 2 of those subjects and on OGTT results |

*Abbreviations:* *T2D: Type 2 diabetes, GI: Glucose intolerance, NIDD: non-insulin dependent diabetes mellitus, GA: Glucose Abnormality, DM: diabetes mellitus, IGT: Impaired glucose intolerance*
