# Supplementary material for: Models Predicting Postpartum Glucose Intolerance Among Women with a History of Gestational Diabetes Mellitus: a Systematic Review
Source: Curr Diab Rep. 2023 Jun 9;23(9):231–43. doi: 10.1007/s11892-023-01516-0 (PMC10435618; doi:10.1007/s11892-023-01516-0)
Supplement: Supplementary file 3 — Supplementary file3 (DOCX 16 KB) [file 11892_2023_1516_MOESM3_ESM.docx]

**Table S3. Inclusion and exclusion criteria of studies**

| **Articles** | **Inclusion criteria** | **Exclusion criteria** |
| --- | --- | --- |
| Bengtson 2022 | Postpartum women with recent GDM were recruited between January 2017 and July 2018 and were eligible for enrolment if they were > 18 years of age and fluent in English or Spanish | If they intended to move outside of the state in the coming year, could not tolerate a 75-gram glucose tolerance test (e.g., women with a history of gastric bypass), or had a known hemoglinopathy |
| Man 2021 | Eligibility criteria included an age of at least 25 years, a body-mass index (the weight in kilograms divided by the square of the height in meters) of 24 or higher (22 or higher in Asians), and a plasma glucose concentration of 95 to 125 mg per deciliter (5.3 to 6.9 mmol per liter) in the fasting state (≤125 mg per deciliter in the American Indian clinics) and 140 to 199 mg per deciliter (7.8 to 11.0 mmol per liter) two hours after a 75-g oral glucose load. | If they were taking medicines known to alter glucose tolerance or if they had illnesses that could seriously reduce their life expectancy or their ability to participate in the trial. |
| Bartáková 2021 | participants having GDM (all Caucasian of Czech nationality from South Moravian Region, Czech Republic), who were followed between 2011–2013 and who at the same time underwent repeated oGTT test up to 1 year after delivery | Established diabetes mellitus type 1 or 2 before pregnancy (diagnosed according to recent WHO criteria, non-Caucasian origin, and multiple pregnancies |
| Joglekar 2020 | If they had previous GDM pregnancy, were aged at least 18 years and English speaking and Primigravid | Women with pre-existing diabetes were excluded |
| Muche 2020 | If they were 18 years or older, had singleton pregnancy and at 20-24 weeks gestation during commencement time | Pregnant women who had pre-existing or overt diabetes, chronic diseases, or on medications that might affect glucose metabolism (steroids, Î²-adrenergic agonists, antipsychotic drugs) at commencement were excluded. |
| Khan 2019 | Racially and ethnically diverse group of women, with GDM (age 20 - 45 years), who delivered singleton pregnancies at ≥ 35 weeks of gestation at Kaiser Permanente Northern California (KPNC) hospitals between 2008 and 2011 | Those women who are diagnosed as diabetes during baseline assessment |
| Kondo 2018 | Women with confirmed GDM were included | Those with known diabetes and overt diabetes were excluded |
| Allalou 2016 | Racially and ethnically diverse women (age 20–45 years) in whom GDM was diaagnosed via a 3-h 100-g OGTT based on the Carpenter and Coustan criteria had no history of diabetes or other serious health conditions, received prenatal care, and delivered singleton pregnancies after $35 weeks of gestation at Kaiser Permanente Northern California (KPNC) hospitals during 2008–2011 | NR |
| Ignell 2016 | Pregnant women with different glycaemic status | Women already diagnosed with diabetes at 1- to 2-year follow-up or later |
| Köhler 2016 | All subjects recruited into the study were newly diagnosed without a history of GDM in previous GDM | Not reported |
| Bartáková 2015 | (i) GDM diagnosis classified by International Classification of Diseases (ICD)-10 code O24.4 or O24.9,  (ii) GDM diagnosed by 3-point 75 g of glucose 2-h oGTT between 24th and 28th week of pregnancy at the University Hospital Brno,  (ii) completed postpartum oGTT 6 weeks up to 1 year after the indexed delivery | (i) pre-gestational type 1 diabetes mellitus (T1DM), T2DM or abnormal glucose tolerance (i.e. O24.0– O24.3 or R73) and  (ii) GDM diagnosed by oGTT outside the University Hospital Brno. |
| Lappas 2015 | If they had a previous GDM pregnancy, were aged at least 18 years and English speaking. | Women with pre-existing diabetes were excluded |
| Cormier 2015 | Women living in the greater Quebec City area, aged ≥18 years, with a pregnancy between 2003 and 2010 were invited to participate. | Women were excluded if they were pregnant at the time of the study or if they had type-I diabetes. |
| Kwak 2012 | Women diagnosed with GDM | Women who had diabetes before pregnancy or positive results for GAD antibodies were excluded from the study |
| Kjos SL1995 | Those who completed postpartum oGTT 6 weeks up to 1 year after the indexed delivery." | Women with pre-existing diabetes were excluded. |

*Abbreviations: GDM: gestational diabetes mellitus, NR: not reported, OGTT: oral glucose tolerance test, T1DM: type 1 diabetes mellitus, WHO: world health organizations*
