# Supplementary material for: Models Predicting Postpartum Glucose Intolerance Among Women with a History of Gestational Diabetes Mellitus: a Systematic Review
Source: Curr Diab Rep. 2023 Jun 9;23(9):231–43. doi: 10.1007/s11892-023-01516-0 (PMC10435618; doi:10.1007/s11892-023-01516-0)
Supplement: Supplementary file 4 — Supplementary file4 (DOCX 29 KB) [file 11892_2023_1516_MOESM4_ESM.docx]

**Title:** Risk Prediction models predicting postpartum glucose intolerance among women who had gestational diabetes mellitus: Systematic review

**FINAL SERACHING METHODS AND RESULTS**

| **Medline Searching Methods** |  |  |
| --- | --- | --- |
| **Risk prediction model** | **Gestational diabetes mellitus** | **Glucose intolerance** |
| Medline |  |  |
| Subject Headings |  |  |
| (exp Artificial Intelligence/ or exp Models, Biological/ or exp Clinical Decision Rules/ or exp Decision Making/ or exp Decision Support Techniques/ or exp Neural Networks, Computer/ or exp Machine Learning/ or exp Models, Statistical/ or exp nomogram/)  AND  (predict* or prognos?s).mp. | Diabetes, Gestational/ | Glucose Intolerance/ Glucose Tolerance Test/ |
| Text words |  |  |
| (predict* adj4 (disease* or biomarker*)).mp.  (predict* adj3 (tool? Or model* or risk or factor? Or score? Or variable? Or rule? Or index )).mp.  (risk? Adj2 (stratification or classification or factor? Or assess* or score? Or tool? Or model?)).mp. | ((gestation* or pregnan*) adj3 (diabet* or T2DM or NIDDM)).mp. | (glucose intolerance* or IGT or Impaired fasting glucose* or glucose tolerance or IFG or Prediabet* or dysglyc?emi*).mp. |
|  |  |  |
|  |  |  |

**OVID Medline search results**

| **#** | **Query** | **Results from 13 May 2022** |
| --- | --- | --- |
| 1 | (exp Artificial Intelligence/ or exp Models, Biological/ or exp Clinical Decision Rules/ or exp Decision Making/ or exp Decision Support Techniques/ or exp Neural Networks, Computer/ or exp Machine Learning/ or exp Models, Statistical/ or exp nomogram/) and (predict* or prognos?s).tw. | 283,152 |
| 2 | (predict* adj4 (disease* or biomarker*)).tw. | 75,827 |
| 3 | (predict* adj3 (tool? or model* or risk or factor? or score? or variable? or rule? or index)).tw. | 353,325 |
| 4 | (risk? adj2 (stratification or classification or factor? or assess* or score? or tool? or model?)).tw. | 857,576 |
| 5 | 1 or 2 or 3 or 4 | 1,370,802 |
| 6 | exp Diabetes, Gestational/ | 15,678 |
| 7 | ((gestation* or pregnan*) adj3 (diabet* or T2DM or NIDDM)).tw. | 25,294 |
| 8 | 6 or 7 | 28,444 |
| 9 | exp Glucose Intolerance/ | 9,567 |
| 10 | exp Glucose Tolerance Test/ | 36,593 |
| 11 | (glucose intolerance* or IGT or Impaired fasting glucose* or glucose tolerance or IFG or Prediabet* or dysglyc?emi*).tw. | 67,855 |
| 12 | 9 or 10 or 11 | 83,051 |
| 13 | 5 and 8 and 12 | 1,473 |
| 14 | limit 13 to english language | 1,373 |

**OVID Embase search results**

| **#** | **Query** | **Results from 13 May 2022** |
| --- | --- | --- |
| 1 | (exp Artificial Intelligence/ or exp Models, Biological/ or exp Clinical Decision Rules/ or exp Decision Making/ or exp Decision Support Techniques/ or exp Neural Networks, Computer/ or exp Machine Learning/ or exp Models, Statistical/ or exp nomogram/) and (predict* or prognos?s).tw. | 356,745 |
| 2 | (predict* adj4 (disease* or biomarker*)).tw. | 120,724 |
| 3 | (predict* adj3 (tool? or model* or risk or factor? or score? or variable? or rule? or index)).tw. | 499,726 |
| 4 | (risk? adj2 (stratification or classification or factor? or assess* or score? or tool? or model?)).tw. | 1,261,498 |
| 5 | 1 or 2 or 3 or 4 | 1,968,025 |
| 6 | exp Diabetes, Gestational/ | 44,504 |
| 7 | ((gestation* or pregnan*) adj3 (diabet* or T2DM or NIDDM)).tw. | 39,257 |
| 8 | 6 or 7 | 53,385 |
| 9 | exp Glucose Intolerance/ | 20,444 |
| 10 | exp Glucose Tolerance Test/ | 73,433 |
| 11 | (glucose intolerance* or IGT or Impaired fasting glucose* or glucose tolerance or IFG or Prediabet* or dysglyc?emi*).tw. | 103,109 |
| 12 | 9 or 10 or 11 | 137,344 |
| 13 | 5 and 8 and 12 | 2,560 |
| 14 | limit 13 to english language | 2,374 |
|  |  |  |
|  |  |  |
|  |  |  |

| **Scopus Searching methods** |  |  |
| --- | --- | --- |
| (“Artificial Intelligence” or “Models, Biological” or “Clinical Decision Rules” or “Decision Making” or “Decision Support Techniques” or “Neural Networks, Computer” or “Machine Learning” or “Models, Statistical” or nomogram)  AND  (predict* or prognos?s) | ((gestation* or pregnan*) W/3 (diabet* or T2DM or NIDDM)) | (glucose intolerance* or IGT or Impaired fasting glucose* or glucose tolerance or IFG or Prediabet* or dysglyc?emi*) |
| (predict* W/4 (disease* or biomarker*))  OR  (predict* W/3 (tool? Or model* or risk or factor? Or score? Or variable? Or rule? Or index ))  OR  (risk? W/2 (stratification or classification or factor? Or assess* or score? Or tool? Or model?)) |  |  |
|  |  |  |
|  |  |  |
| **Web of Science Searching methods** |  |  |
| (“Artificial Intelligence” or “Models, Biological” or “Clinical Decision Rules” or “Decision Making” or “Decision Support Techniques” or “Neural Networks, Computer” or “Machine Learning” or “Models, Statistical” or nomogram)  AND  (predict* or prognos?s) | ((gestation* or pregnan*) NEAR/3 (diabet* or T2DM or NIDDM)) | (glucose intolerance* or IGT or Impaired fasting glucose* or glucose tolerance or IFG or Prediabet* or dysglyc?emi*) |
| (predict* NEAR/4 (disease* or biomarker*))  OR  (predict* NEAR/3 (tool? Or model* or risk or factor? Or score? Or variable? Or rule? Or index ))  OR  (risk? NEAR/2 (stratification or classification or factor? Or assess* or score? Or tool? Or model?)) |  |  |
|  |  |  |
|  |  |  |

| CINHAL SEARCHING METHODS |  |  |
| --- | --- | --- |
| Risk Prediction Model* | Gestational Diabetes Mellitus | Glucose Intolerance |
|  | Subject headings |  |
| (“Artificial Intelligence” or “Models, Biological” or “Clinical Decision Rules” or “Decision Making” or “Decision Support Techniques” or “Neural Networks, Computer” or “Machine Learning” or “Models, Statistical” or nomogram)  AND  (predict* or prognos?s) | [Diabetes Mellitus, Gestational](javascript:XslPostBack('ctl00$ctl00$MainContentArea$MainContentArea$ctrlResults','meshList','index%7C4%24term%7CDiabetes%20Mellitus%2C%20Gestational%24cmd%7CmeshList');) | (glucose intolerance* or IGT or Impaired fasting glucose* or glucose tolerance or IFG or Prediabet* or dysglyc?emi*) |
| (predict* N4 (disease* or biomarker*))  OR  (predict* N3 (tool? Or model* or risk or factor? Or score? Or variable? Or rule? Or index ))  OR  (risk? N2 (stratification or classification or factor? Or assess* or score? Or tool? Or model?)) | [Pregnancy in Diabetes](https://web.p.ebscohost.com/ehost/mesh/tree?term=Pregnancy%20in%20Diabetes&sid=912c77d8-2886-4737-81bc-0ef9e1b6f6d7%40redis&vid=7) |  |
|  |  |  |
|  | ((gestation* or pregnan*) N3 (diabet* or T2DM or NIDDM)) |  |
|  |  |  |
|  |  |  |

**Total search results imported to Endnote for deduplication**

| R.N | Date of searching | Database | Final number of results |
| --- | --- | --- | --- |
| 1 | May 12, 2022 | Ovid-Medline | 1377 |
| 2 | May 12, 2022 | Embase | 2374 |
| 3 | May 12, 2022 | Maternity & Infant Care Database (MIDIRS) | 349 |
| 4 | May 12, 2022 | Global Health 1910 to 2022 Week 18 | 576 |
| 5 | May 12, 2022 | Scopus | 71 |
| 6 | May 12, 2022 | Web of Science | 366 |
| 7 | May 12,2022 | EMCARE (OVID) | 628 |
| 8 | May 13,2022 | CINHAL | 1042 |
|  |  | Total search results | 6783 |

**Endnote software**

| Total imported to endnote | Removed by endnote automatically | Eligible for deduplication | Duplication removed | Removed for irrelevant or some reasons | Total imported for Covidence |
| --- | --- | --- | --- | --- | --- |
| 6783 | 12 | 6771 | 3264 | 4 | 3506 |
|  |  |  |  |  |  |

**COVIDENCE Software**

| Total imported for Covidence | System and manual duplicates removed | Total  Eligible for title and abstract screening | Excluded by title and abstract screening | Eligible for full text review |
| --- | --- | --- | --- | --- |
| 3506 | 51 | 3455 | 3402 | 53 |
|  |  |  |  |  |
