# Supplementary material for: Models Predicting Postpartum Glucose Intolerance Among Women with a History of Gestational Diabetes Mellitus: a Systematic Review
Source: Curr Diab Rep. 2023 Jun 9;23(9):231–43. doi: 10.1007/s11892-023-01516-0 (PMC10435618; doi:10.1007/s11892-023-01516-0)
Supplement: Supplementary file 5 — Supplementary file5 (DOCX 17 KB) [file 11892_2023_1516_MOESM5_ESM.docx]

**Extraction checklist**

**Title:**

1. Study ID (**First Author and year**):
2. Country in which data was gathered (**Name of the country**):
3. Ethics approval obtained (**Yes/No**):
4. Setting (**clinical(hospital-based), non-clinical (community based) or mixed**):
5. Data source (**Electronic health records (EHRs) and questionnaires**)):
6. Study design (**Retrospective cohort, cross-sectional, prospective cohort**):
7. Included cases (**GDM patients**):

Inclusion criteria:

Exclusion criteria:

1. GDM diagnosis criteria (**NDDG, NR, IADPSG, Carpenter-Coustan thresholds, WHO, ADA, ICD-9-CM diagnostic codes, ADIPS, FPG: 5. 1 mmol /L， OGTT 1 h:10. 0mmol /L， OGTT 2h: 8. 5 mmol /L), FPG: 5.5 mmol⁄L or a 2-h level of 8.0 mmol⁄L, CDA**):
2. Sample size determination method explained (**Yes/No**):
3. Number of participants included:
4. Selection method **(census, sampling):**
5. Primary outcome definition (diabetes, non-insulin dependent diabetes mellitus(NIDDM), glucose abnormality, Glucose intolerance Impaired glucose tolerance, dysglycemia, pre-diabetics)
6. Glucose intolerance diagnosis method (**Outcome diagnosis criteria) (NDDG, NR, IADPSG, Carpenter-Coustan thresholds, WHO, ADA, ICD-9-CM diagnostic codes, ADIPS, FPG: 5. 1 mmol /L， OGTT 1 h:10. 0 mmol /L， OGTT 2h: 8. 5 mmol /L), FPG: 5.5 mmol⁄L or a 2-h level of 8.0 mmol⁄L, CDA**):
7. Prediction temporality (**prognostic, diagnostic**):
8. Postpartum Glucose Intolerance test time:
9. Prevalence of outcome (Number of participants with outcomes):
10. Type of predictors used (**socio-demographic characteristics, maternal factors, neonatal factors, biochemical characteristics, lipid profiles, genetic characteristics**), **demographic profile, obstetric history, anthropometric measurements, type of treatment of GDM (diet or insulin), behavioural factors (alcohol use and coffee intake), lifestyle parameters (dietary diversity and physical activity), antenatal depression status and blood glucose value (fasting plasma glucose (FPG) and OGTT).**
11. List of candidate predictors included (**include all individual predictors**) demographic profile, obstetric history, anthropometric measurements, type of treatment of GDM (diet or insulin), behavioural factors (alcohol use and coffee intake), lifestyle parameters (dietary diversity and physical activity), antenatal depression status and blood glucose value (fasting plasma glucose (FPG) and OGTT). All participants also had FPG and 2-hour OGTT blood
12. Pre-processing described (Yes/No):
13. Missing data described (Yes/No):
14. Missingness handling described (**Imputation of missing data**,):
15. Method for selection of predictors for inclusion in multivariable modelling, Variable selection method (Feature selection algorithms) Feature selection described **(Logistic regression, Information value, Multivariable logistic regression with backward stepwise elimination, Univariate significance level, clinical importance, multivariate logistic regression, Multivariate logistic regression, Univariate backward selection; XGBoost, Univariate logistic regression, Multivariate model, RECPAM):** **p≤0.2 in the univariable analysis**
16. Method for selection of predictors during multivariable modelling like full model approach, backward or forward selection and criteria used (e.g., p-value, Akaike Information Criterion):
17. Statistical/data analysis methods used Data analysis/modelling method used (**Non-Logistic regression Method used (machine learning), Statistical/data analysis methods used:**
18. **Shrinkage of predictor weights or regression coefficients** (e.g., no shrinkage, uniform shrinkage, penalized estimation) Variables included in final model (Number of predictors in the final model including interactions (Predictors in final model including interactions): Advanced maternal age, High fasting plasma glucose level at diagnosis, Overweight and/or obesity, and Antenatal depression
19. Overall model performance measures

R-squard

Brier score

other (specify)

1. Calibration yes/no

calibration plot (judgment by authors (e.g. good, moderate))

calibration table (% over-/underprediction (similar to observed/expected ratio))

calibration slope

calibration intercept

Hosmer-Lemeshow test:

observed/expected ratio

other

1. Discrimination
   1. c-statistic
   2. AUC:
   3. D-statistic
   4. log-rank
   5. Sensitivity specificity PPV NPV
   6. other model evaluation metrics (specify)
2. Internal validation (Yes/No)

discrimination

Calibration

1. External validation (Yes/No)

discrimination

Calibration

1. Mode of model presentation: Reporting method: Risk score Full regression formula (beta's/coefficients + intercept/baseline hazard, overall or of example patient) Full regression formula (beta's/coefficients + intercept/baseline hazard, overall or of example patient) partial regression formula (hazard/odd ratio's, no intercept/baseline hazard) sum score/chart nomogram, predictions for specific risk subgroups, online tool, partial regression formula (hazard/odd ratio's, no intercept/baseline hazard), sum score/chart nomogram, predictions for specific risk subgroups online tool other (specify):
2. Funding:
